# Supplementary material for: Resolving the full spectrum of human genome variation using Linked-Reads
Source: Genome Res. 2019 Apr;29(4):635–45. doi: 10.1101/gr.234443.118 (PMC6442396; doi:10.1101/gr.234443.118)
Supplement: Supplemental Material [file supp_gr.234443.118_Supplemental_longranger-2.2.zip › longranger-2.2.tar.bz2!/lr2.2-public/lib/stan/cmdstan/stan/doc/api/html/base__static__hmc_8hpp_source.html]

Stan: src/stan/mcmc/hmc/static/base\_static\_hmc.hpp Source File


|  |  |
| --- | --- |
|  | Stan  2.5.0  probability, sampling & optimization |


- Main Page
- Related Pages
- Namespaces
- Classes
- Files

- File List
- File Members

All Classes Namespaces Files Functions Variables Typedefs Enumerations Enumerator Friends Macros Pages

- src
- stan
- mcmc
- hmc
- static

base\_static\_hmc.hpp

Go to the documentation of this file.

1 #ifndef STAN\_\_MCMC\_\_BASE\_\_STATIC\_\_HMC\_\_BETA

2 #define STAN\_\_MCMC\_\_BASE\_\_STATIC\_\_HMC\_\_BETA

3

4 #include <math.h>

5 #include <boost/math/special\_functions/fpclassify.hpp>

6 #include <stan/mcmc/hmc/base\_hmc.hpp>

7 #include <stan/mcmc/hmc/hamiltonians/ps\_point.hpp>

8

9 namespace stan {

10

11  namespace mcmc {

12

13  // Hamiltonian Monte Carlo

14  // with static integration time

15

16  template <class M, class P, template<class, class> class H,

17  template<class, class> class I, class BaseRNG>

18  class base\_static\_hmc: public base\_hmc<M, P, H, I, BaseRNG> {

19

20  public:

21

22  base\_static\_hmc(M &m, BaseRNG& rng, std::ostream\* o, std::ostream\* e):

23  base\_hmc<M, P, H, I, BaseRNG>(m, rng, o, e), T\_(1)

24  { update\_L\_(); }

25

26  ~base\_static\_hmc() {};

27

28  sample transition(sample& init\_sample) {

29

30  this->sample\_stepsize();

31

32  this->seed(init\_sample.cont\_params());

33

34  this->hamiltonian\_.sample\_p(this->z\_, this->rand\_int\_);

35  this->hamiltonian\_.init(this->z\_);

36

37  ps\_point z\_init(this->z\_);

38

39  double H0 = this->hamiltonian\_.H(this->z\_);

40

41  for (int i = 0; i < L\_; ++i)

42  this->integrator\_.evolve(this->z\_, this->hamiltonian\_, this->epsilon\_);

43

44  double h = this->hamiltonian\_.H(this->z\_);

45  if (boost::math::isnan(h)) h = std::numeric\_limits<double>::infinity();

46

47  double acceptProb = std::exp(H0 - h);

48

49  if (acceptProb < 1 && this->rand\_uniform\_() > acceptProb)

50  this->z\_.ps\_point::operator=(z\_init);

51

52  acceptProb = acceptProb > 1 ? 1 : acceptProb;

53

54  return sample(this->z\_.q, - this->hamiltonian\_.V(this->z\_), acceptProb);

55

56  }

57

58  void write\_sampler\_param\_names(std::ostream& o) {

59  o << "stepsize\_\_,int\_time\_\_,";

60  }

61

62  void write\_sampler\_params(std::ostream& o) {

63  o << this->epsilon\_ << "," << this->T\_ << ",";

64  }

65

66  void get\_sampler\_param\_names(std::vector<std::string>& names) {

67  names.push\_back("stepsize\_\_");

68  names.push\_back("int\_time\_\_");

69  }

70

71  void get\_sampler\_params(std::vector<double>& values) {

72  values.push\_back(this->epsilon\_);

73  values.push\_back(this->T\_);

74  }

75

76  void set\_nominal\_stepsize\_and\_T(const double e, const double t) {

77  if(e > 0 && t > 0) {

78  this->nom\_epsilon\_ = e;

79  T\_ = t;

80  update\_L\_();

81  }

82  }

83

84  void set\_nominal\_stepsize\_and\_L(const double e, const int l) {

85  if(e > 0 && l > 0) {

86  this->nom\_epsilon\_ = e;

87  L\_ = l;

88  T\_ = this->nom\_epsilon\_ \* L\_; }

89  }

90

91  void set\_T(const double t) {

92  if(t > 0) {

93  T\_ = t;

94  update\_L\_();

95  }

96  }

97

98  void set\_nominal\_stepsize(const double e) {

99  if(e > 0) {

100  this->nom\_epsilon\_ = e;

101  update\_L\_();

102  }

103  }

104

105  double get\_T() { return this->T\_; }

106  int get\_L() { return this->L\_; }

107

108  protected:

109

110  double T\_;

111  int L\_;

112

113  void update\_L\_() {

114  L\_ = static\_cast<int>(T\_ / this->nom\_epsilon\_);

115  L\_ = L\_ < 1 ? 1 : L\_;

116  }

117

118  };

119

120  } // mcmc

121

122 } // stan

123

124

125 #endif

stan::mcmc::base\_static\_hmc::write\_sampler\_param\_names

void write\_sampler\_param\_names(std::ostream &o)

**Definition:** base\_static\_hmc.hpp:58

boost::math::isnan

bool isnan(const stan::agrad::var &v)

Checks if the given number is NaN.

**Definition:** boost\_fpclassify.hpp:69

stan::mcmc::base\_hmc::z\_

P z\_

**Definition:** base\_hmc.hpp:133

stan::mcmc::base\_static\_hmc::write\_sampler\_params

void write\_sampler\_params(std::ostream &o)

**Definition:** base\_static\_hmc.hpp:62

base\_hmc.hpp

stan::mcmc::base\_static\_hmc::set\_nominal\_stepsize\_and\_T

void set\_nominal\_stepsize\_and\_T(const double e, const double t)

**Definition:** base\_static\_hmc.hpp:76

stan::mcmc::base\_hmc::hamiltonian\_

H< M, BaseRNG > hamiltonian\_

**Definition:** base\_hmc.hpp:135

stan::mcmc::ps\_point

**Definition:** ps\_point.hpp:16

stan::mcmc::base\_static\_hmc::set\_nominal\_stepsize\_and\_L

void set\_nominal\_stepsize\_and\_L(const double e, const int l)

**Definition:** base\_static\_hmc.hpp:84

stan::mcmc::base\_hmc::sample\_stepsize

void sample\_stepsize()

**Definition:** base\_hmc.hpp:125

stan::mcmc::base\_static\_hmc::L\_

int L\_

**Definition:** base\_static\_hmc.hpp:111

stan::mcmc::base\_static\_hmc::set\_nominal\_stepsize

void set\_nominal\_stepsize(const double e)

**Definition:** base\_static\_hmc.hpp:98

stan::mcmc::sample::cont\_params

double cont\_params(int k) const

**Definition:** sample.hpp:30

stan::mcmc::base\_static\_hmc::set\_T

void set\_T(const double t)

**Definition:** base\_static\_hmc.hpp:91

ps\_point.hpp

stan::mcmc::base\_hmc::nom\_epsilon\_

double nom\_epsilon\_

**Definition:** base\_hmc.hpp:142

stan::mcmc::base\_hmc::rand\_int\_

BaseRNG & rand\_int\_

**Definition:** base\_hmc.hpp:137

stan::mcmc::base\_static\_hmc

**Definition:** base\_static\_hmc.hpp:18

stan::mcmc::base\_hmc

**Definition:** base\_hmc.hpp:20

stan::mcmc::base\_static\_hmc::base\_static\_hmc

base\_static\_hmc(M &m, BaseRNG &rng, std::ostream \*o, std::ostream \*e)

**Definition:** base\_static\_hmc.hpp:22

stan::math::e

double e()

Return the base of the natural logarithm.

**Definition:** constants.hpp:86

stan::mcmc::base\_static\_hmc::~base\_static\_hmc

~base\_static\_hmc()

**Definition:** base\_static\_hmc.hpp:26

stan::mcmc::base\_static\_hmc::get\_L

int get\_L()

**Definition:** base\_static\_hmc.hpp:106

stan::mcmc::base\_hmc::seed

void seed(const Eigen::VectorXd &q)

**Definition:** base\_hmc.hpp:51

stan::mcmc::base\_static\_hmc::get\_T

double get\_T()

**Definition:** base\_static\_hmc.hpp:105

stan::mcmc::base\_hmc::rand\_uniform\_

boost::uniform\_01< BaseRNG & > rand\_uniform\_

**Definition:** base\_hmc.hpp:140

stan::mcmc::base\_static\_hmc::get\_sampler\_param\_names

void get\_sampler\_param\_names(std::vector< std::string > &names)

**Definition:** base\_static\_hmc.hpp:66

stan::mcmc::base\_static\_hmc::transition

sample transition(sample &init\_sample)

**Definition:** base\_static\_hmc.hpp:28

stan::mcmc::base\_static\_hmc::T\_

double T\_

**Definition:** base\_static\_hmc.hpp:110

stan::mcmc::base\_static\_hmc::update\_L\_

void update\_L\_()

**Definition:** base\_static\_hmc.hpp:113

stan::agrad::exp

fvar< T > exp(const fvar< T > &x)

**Definition:** exp.hpp:16

stan::mcmc::sample

**Definition:** sample.hpp:13

stan::common::sample

void sample(stan::mcmc::base\_mcmc \*sampler, int num\_warmup, int num\_samples, int num\_thin, int refresh, bool save, stan::io::mcmc\_writer< Model, SampleRecorder, DiagnosticRecorder, MessageRecorder > &writer, stan::mcmc::sample &init\_s, Model &model, RNG &base\_rng, const std::string &prefix, const std::string &suffix, std::ostream &o, StartTransitionCallback &callback)

**Definition:** sample.hpp:13

stan::mcmc::base\_static\_hmc::get\_sampler\_params

void get\_sampler\_params(std::vector< double > &values)

**Definition:** base\_static\_hmc.hpp:71

stan::mcmc::base\_hmc::integrator\_

I< H< M, BaseRNG >, P > integrator\_

**Definition:** base\_hmc.hpp:134

stan::mcmc::base\_hmc::epsilon\_

double epsilon\_

**Definition:** base\_hmc.hpp:143


---

[ Stan Home Page ]

*© 2011–2014,
Stan Development Team.*
